# Fluorescent Dye-Doped Brightening Polymer-Stabilized Bistable Cholesteric Liquid Crystal Films

**DOI:** 10.3390/molecules28083509

**Published:** 2023-04-16

**Authors:** Yuzhen Zhao, Chaonian Li, Tingting Lang, Yitian Sun, Qingbo Li, Xinli Shi, Ruijuan Yao, Huimin Zhang, Yang Zhao

**Affiliations:** 1 Xi’an Key Laboratory of Advanced Photo-Electronics Materials and Energy Conversion Device, School of Electronic Information, Xijing University, Xi’an 710123, China; 2 Shandong Lanbeisite Educational Equipment Group Co., Ltd., Jinan 250100, China

**Keywords:** bistable cholesteric liquid crystal, organic fluorescent dyes, high contrast ratio, brightening film

## Abstract

Brightening polymer-stabilized bistable cholesteric liquid crystal (PSBCLC) films with doped fluorescent dyes were prepared using the polymerization-induced phase separation (PIPS) method. The transmittance performance behavior of these films in both states (focal conic and planar) and absorbance change in multiple dye concentrations were studied using a UV/VIS/NIR spectrophotometer. The change occurring in dye dispersion morphology with different concentrations was obtained by means of the polarizing optical microscope. The maximum fluorescence intensity of different dye-doped PSBCLC films was measured using a fluorescence spectrophotometer. Moreover, the contrast ratios and driving voltages of these films were calculated and recorded to demonstrate film performance. Finally, the optimal concentration of dye-doped PSBCLC films with a high contrast ratio and a relatively low drive voltage was found. This is expected to have great potential applications in cholesteric liquid crystal reflective displays.

## 1. Introduction

Polymer-stabilized bistable cholesteric liquid crystal (PSBCLC) is generally formed by dispersing photo-polymerization of a prepolymer into the bistable cholesteric liquid crystal (BCLC) matrix [1,2,3,4]. The crosslinked polymer obtained from prepolymer is used to stabilize/lock the oriented liquid crystal molecules and to reduce the switching time and driving voltage [5,6]. In addition, the polymer networks in PSBCLC have a significant effect on the transmittance, response time, absorbance, and dielectric properties of the films [7,8,9]. Therefore, the PSBCLC has received extensive attention, especially in the field of bistable cholesteric liquid crystal reflective displays [10,11,12].

PSBCLC has always attracted much worldwide attention since its discovery because its display devices do not require a high-power backlight and are readable under sunlight [13,14,15,16]. With the continuous development of reflective display technology, a large number of researchers have focused on PSBCLC. Rajendra et al. incorporated dichroic dyes into polymer-dispersed liquid crystal (PDLC) to study the microstructure, optoelectronic properties, and dielectric properties of the composite system [17]. Jae-Won Huh et al. used a dye-doped BCLC to prepare a bistable light shutter in order to reduce power consumption by light shutters [18]. Vijay Kumar Baliyan et al. prepared electrically switchable smart windows by combining two different approaches of doping dichroic dye and increasing film thickness to enhance shielding performance in the voltage-off state [19]. However, few studies have focused on improving the utilization of incident lights, which is one of the difficulties faced by reflective bistable cholesteric liquid crystal displays.

The reflection bandwidth (Δλ) of cholesteric liquid crystal is greatly limited by the helical pitch length (p) and the average refractive index (Δn), according to the equation of Δλ = Δn p. Therefore, only a few incident lights can be reflected by cholesteric liquid crystal displays. In order to improve the utilization of incident lights, two fluorescent dyes (perylene and diisobutyl perylenedicarboxylate) were selected to absorb incident lights out of the reflection bandwidth (Δλ) and respectively doped into PSBCLC films. Dye-doped PSBCLC films of different concentrations were prepared to investigate the performance change in their microscopic morphologies and optical properties. Furthermore, the drive voltage from the planar state to the focal conic state was measured to evaluate the energy consumption of dye-doped PSBCLC films. Finally, the fluorescent dye-doped PSBCLC films with high brightness, high contrast ratio, and low energy consumption were prepared, which had great significance in the devices of electronic books and papers, light shutters, and LCD tablets. 

## 2. Results and Discussion

### 2.1. Building the Best PSBCLC Polymer Network

**Figure 1:**
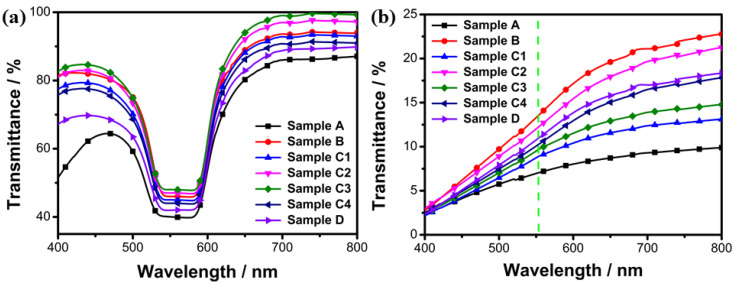
Transmission spectra of seven PSBCLC samples in (**a**) planar states; (**b**) focal conic states.

Polymer networks for anchoring and supporting liquid crystal molecules are very important for PSBCLC, so it was necessary to construct a good polymer network. As shown in Table 1, seven samples were used to explore the optimal polymer network because the prepolymer formula and polymerization intensity directly affect the density and mesh size of the polymer networks [20]. In order to study the effect of polymerization light intensity on the polymer networks, samples C1 to C4 were placed under 1, 2, 3, and 4 mW/cm^2^ of 365 nm UV lights to polymerize for 10 min. Samples A, B, C3, and D were placed at 3 mW/cm^2^ of 365 nm UV lights to study the effect of the prepolymer formula on the polymer networks. Figure 1a shows the transmission spectra of the samples in the planar state. Obviously, sample C3 had the highest transmittance among C1 to C4, because its suitable mesh, formed at a suitable light intensity, reduced the light scattering caused by polymer networks [21,22]. Then, comparing the four samples, A, B, C3, and D, it could be concluded that the higher the prepolymer concentration, the lower the transmittance in the planar state because the polymer networks of the high-concentration prepolymer were very dense and greatly enhanced the scattering of lights. Figure 1b demonstrates the transmission spectra of the samples in the focal conic states. The transmittance of the samples dropped sharply, compared with the samples in the planar state, because of strong light scattering. In the focal cone state, the dye molecules and LC molecules were randomly oriented, leading to an isotropic absorption and a strong light-scattering state of the window. In the planar state, the LC had no scattered light, and the dye molecules were all in a vertical plane, leading to a slightly higher absorption and increased transmittance of the LC cell. The difference in transmittance could effectively increase the contrast ratio and contribute to the PSBCLC display.

**Figure 2:**
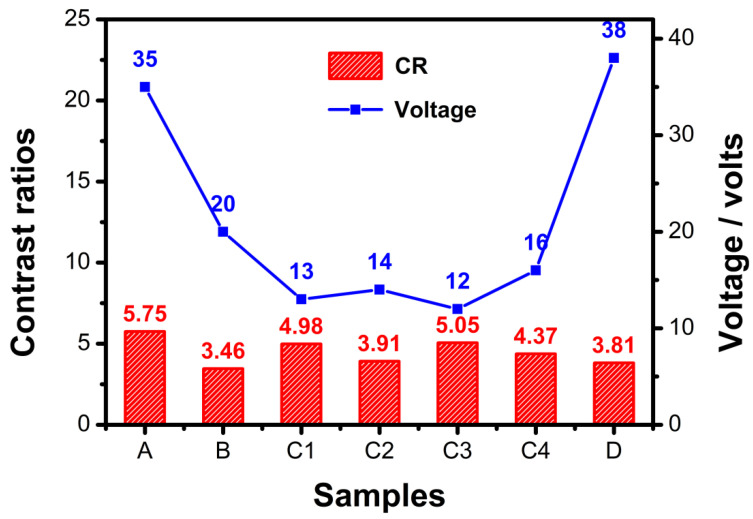
Contrast ratio and driving voltage of seven PSCLC samples.

**Figure 3:**
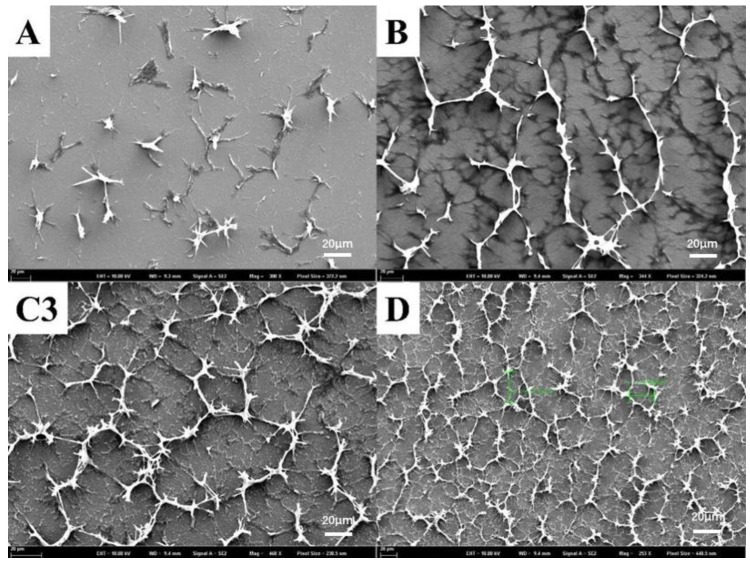
SEM images of PSBCLC samples with different chemical compositions (Samples A, B, C3, and D).

Figure 2 summarizes the contrast ratios (CRs) and the driving voltage from the planar state to the focal conic state. The CRs were calculated using the transmittance ratio of the planar state divided by the focal state at 555 nm. As shown in Figure 2, sample A had the highest contrast ratio, but its driving voltage was also the highest. For the other six samples, sample C3 had the highest transmittance and the lowest driving voltage, so it could easily be concluded that the best PSBCLC was sample C3. Figure 3 shows the SEM images of samples A, B, C3, and D after the removal of liquid crystals, which microscopically showed the effect of the prepolymer ratio on the film morphology directly. Comparing sample A and sample D, when the content of the monofunctional monomer IBOA was too much higher than that of the bifunctional monomer C6M, a better network could not be formed. In B, C3, and D, the increased content of the prepolymer formed a dense network, and sample C3 exhibited a homogeneous morphology with a suitable network size, which matched its electro-optical properties, as shown in Figure 2.

### 2.2. Study on Photoelectric Properties of Fluorescent Dye-Doped Liquid Crystal Composite System

#### 2.2.1. Dispersion of Fluorescent Dyes in Liquid Crystal Systems

**Figure 4:**
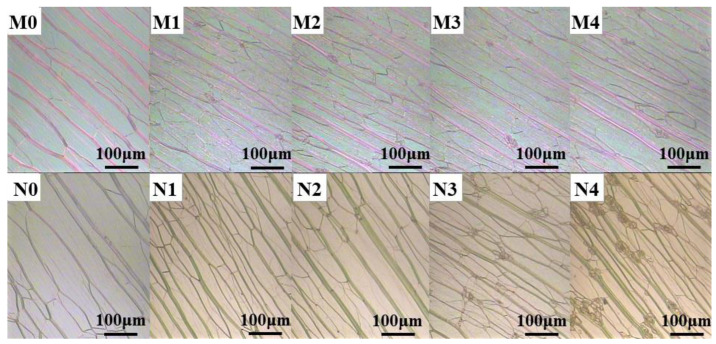
POM images of two dye-doped systems: M0–M4 (0.0 wt% dye1, 0.5 wt% dye1, 1.0 wt% dye1, 1.5 wt% dye1, 2.0 wt% dye1) and N0–N4 (0.0 wt% dye2, 0.5 wt% dye2, 1.0 wt% dye2, 1.5 wt% dye2, 2.0 wt% dye2) were polymer-stabilized films modulated about the compound ratios of C3 samples.

Figure 4 shows the planar texture photos of two dye-doped systems observed with a polarizing microscope. M0–M4 and N0–N4 were polymer-stabilized films modulated about the compound ratios of C3 samples. The differences among them were the different reflection bands of the chiral nematic phase liquid crystals in the M and N groups, as well as the difference in the ratio of liquid crystals within each group. It could be clearly seen that the fluorescent dyes were distributed at the defects of the liquid crystal (LC). When the dyes were doped at a low concentration, the dye molecules were uniformly dispersed in the LC systems, and there were no solid fluorescent particles. As the dye concentration increased, there were more and more solid fluorescent particles because of the aggregation effect, which not only enhanced the light scattering of the system but also caused the aggregation-caused quenching (ACQ) effect: the emission of conventional luminophores was often weakened in the solid state in comparison to in the solution, due to aggregate formation in the condensed phase [23]. The organic dyes and many aromatic luminophores suffered from the aggregation-caused quenching (ACQ) effect, as Birks summarized in his book in 1970 [24]. They emitted strong fluorescence as isolated molecules, but the fluorescence was seriously quenched when they were in the aggregate or solid states. This phenomenon was studied, due to the intermolecular π−π stacking interactions by the aromatic rings of the fluorogens, which will exhaust the energy of the molecule in the excited state by the nonradiative channels, resulting in the emission quenching of the luminophores. The so-called ACQ effect is very common.

#### 2.2.2. Effect of Fluorescent Dye Concentration on Transmission Spectra and Driving Voltage

**Figure 5:**
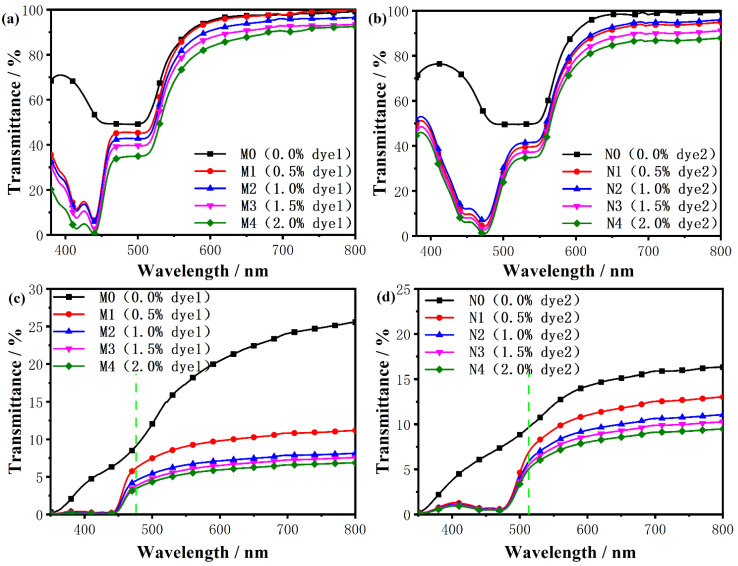
(**a**,**c**) The transmission spectra of different concentrations of dye1-doped PSBCLCs in the planar state and the focal conic state, respectively. (**b**,**d**) The transmission spectra of different concentrations of dye2-doped PSBCLCs in the planar state and the focal conic state, respectively.

The transmittance spectra of different concentrations of dye1-doped and dye2-doped composite systems are shown in Figure 5, respectively. The transmission wavelength of the glass liquid crystal cartridge, which is the carrier vessel of the composite system, decreased violently below 380 nm, so the part of the transmission rate reduction resulting from the glass was removed in the figure. Additionally, the transmittance of the dye-doped system was lower than that of the undoped system in Figure 5a,b, where the transmittance was close to 50%. Meanwhile, the transmittance of the dye-undoped system was also higher than the dye-doped system in the focal conic state by applying the alternating current in Figure 5c,d. In general, the transmittance of the LC composite system gradually decreased as the concentration increased, due to the strong scattering effect of solid fluorescent particles. In addition, the transmittance of the dye-doped composite system in the focal conic state was much lower than in the planar state, due to the strong light scattering of the liquid crystal molecules.

**Figure 6:**
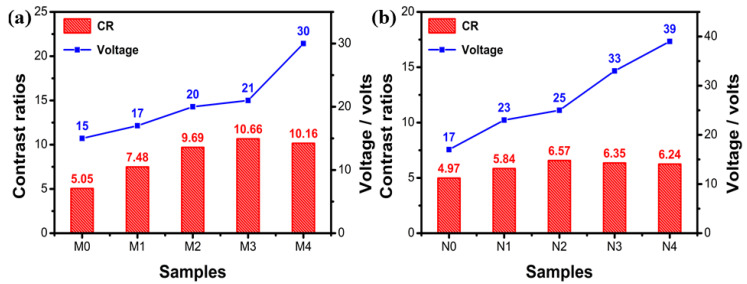
(**a**) Contrast ratios and driving voltages of different concentrations of dye1-doped PSBCLCs; (**b**) contrast ratios and driving voltages of different concentrations of dye2-doped PSBCLCs.

Figure 6a,b depicts the CR and the driving voltage of dye1-doped and dye2-doped PSBCLCs, respectively. The contrast ratios of the samples were calculated using the transmittance ratio of the planar state divided by the focal state at the center of the LC1 (475 nm) and LC2 (515 nm) reflection wavelengths. It clearly demonstrated that the driving voltage of the dye1- and dye2-doped PSBCLCs were gradually increasing with the dye concentration because the dye solid particles increased the motion resistance of the LC molecules, driven by the external electric field. It was clear that the 1.5% wt% dye1-doped system and the 1.0% wt% dye2-doped system had the highest CRs, respectively. As the dye concentration increased, the contrast ratio of the system gradually increased because the emission intensity of the fluorescent dye was much higher than that of the scattering of the solid dye particles. However, when the concentration of the fluorescent dye was too high, the aggregation-caused quenching (ACQ) effect occurred, which resulted in a decrease in the emission intensities of the fluorescent dyes. Therefore, the CRs of the doped system gradually increased before ACQ and gradually decreased after ACQ. Considering both CR and drive voltage, the 1.5 wt% dye1-doped PSBCLC and the 1.0 wt% dye2-doped PSBCLC had the highest CRs and small driving voltages; they were selected as the best fluorescent dye-doped PSBCLCs.

#### 2.2.3. Effect of Fluorescent Dye Concentration on Absorption and Emission Spectra

**Figure 7:**
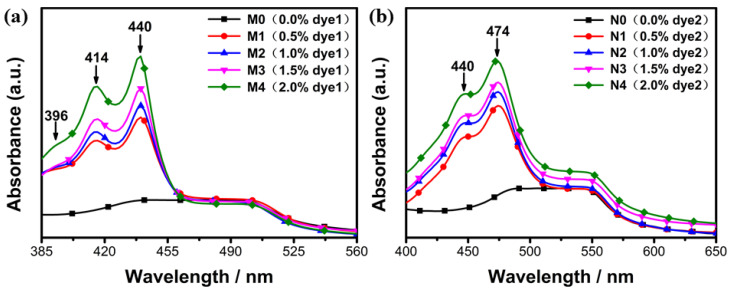
(**a**) The absorption spectra of different concentrations of dye1-doped PSBCLCs; (**b**) the absorption spectra of different concentrations of dye2-doped PSBCLCs.

The absorption spectra of dye1- and dye2-doped LC systems with different concentrations are shown in Figure 7a,b, respectively. Samples M0 and N0 had no absorption peaks in the measured wavelength range because there was no fluorescent dye, while other samples had obvious absorption peaks of fluorescent dyes. It could be clearly seen that dye1 and dye2 contained three and two absorption peaks, which were the excitation peaks of the fluorescence emission spectrum. It is well known that the concentration is closely related to the absorbance, so the greater the concentration of the fluorescent dyes, the stronger the absorption intensity.

**Figure 8:**
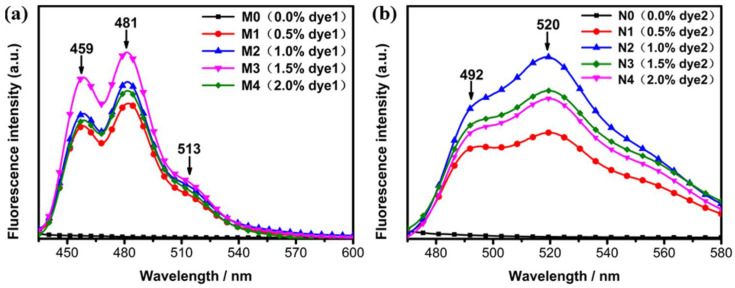
(**a**) The fluorescence emission spectra of different concentrations of dye1-doped PSBCLCs; (**b**) the fluorescence emission spectra of different concentrations of dye2-doped PSBCLCs.

The absorption peaks obtained from the absorption spectra of Figure 8 at 414 nm and 440 nm were respectively used to excite dye1- and dye2-doped systems to obtain the fluorescence emission spectra of Figure 8. It could be seen that there were three emission peaks for dye1 in Figure 8a and two peaks for dye2 in Figure 8b. Since the LC molecules did not emit any lights, samples M0 and N0 had no emission peaks. It could be easily concluded that dye1 mainly emitted blue lights, and dye2 mainly emitted green lights. The fluorescence emission intensity showed a different change as the concentration of the fluorescent dye increased. All the data showed that the optimal doping concentration existed in the fluorescent-doped LC systems, and the optimal doping concentrations of dye1 and dye2 were 1.5 wt% and 1 wt%, respectively. When the doping concentration was lower than the optimal concentration, the fluorescence emission intensity increased with the doping concentration, while when the doping concentration was heavier than the optimal concentration, the fluorescence emission intensity gradually decreased because heavy concentration caused the aggregation-quenching effect of fluorescent dyes.

### 2.3. Mechanism

**Figure 9:**
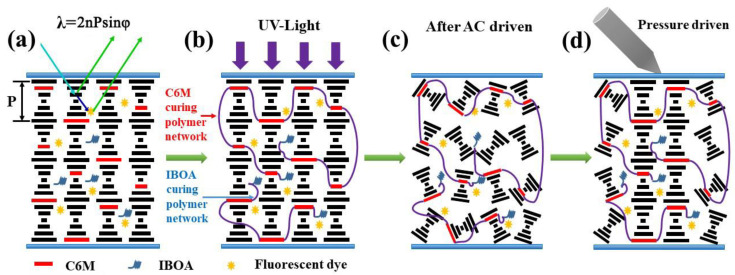
(**a**) Selectively reflect wavelengths of dye-doped PSBCLC film; (**b**) Polymer network of dye-doped PSBCLC film was prepared by the free radical polymerization under 365 nm UV lights; (**c**) The film exhibited the stable focal conic state after AC driven; (**d**) The pressure-sensing region of the film was transformed to planar state after external pressure driven.

Figure 9 vividly depicts the display mechanism of the fluorescent dye-doped PSBCLC film. When the mixed mixture was poured into the prepared LC cell, it was in a stable planar texture that could selectively reflect wavelengths, as shown in Figure 9a. According to Bragg’s law of reflection, λ = nPsinφ, the incident lights that satisfied the law would be reflected, while the other lights would be transmitted, and the maximum selected reflection wavelength could be obtained when the angle φ between the incident light and the liquid crystal surface reached 90° [25,26]. For example, LC2 reflected the wavelengths between 480–550 nm in the planar state without fluorescent dye, and the other wavelengths, except 480–550 nm, were transmitted. When adding the fluorescent dye, the wavelengths between 480–550 nm in the incident lights were still totally reflected, but the transmitted blue lights were absorbed by the fluorescent dye to emit lights in the reflection bandwidth of BCLC. Therefore, the addition of fluorescent dyes increased the utilization of incident lights, greatly enhancing the reflection intensity and effectively improving the contrast ratio and brightness of the PSBCLC film. Figure 9b shows the free radical polymerization of IBOA and C6M monomer prepolymers to form a polymer network under 365 nm UV lights. After the polymerization was completed, a certain alternating current was applied between the LC cells. After removing the alternating current, the film exhibited the stable focal conic state in Figure 9c. Figure 9d illustrates that the liquid crystal in the pressure-sensing region was transformed from the focal conic state to the planar state, driven by external pressure. The incident lights were reflected and transmitted separately in the pressure-driving region and the non-driving region, thus forming a sharp contrast ratio to display an image. 

**Figure 10:**
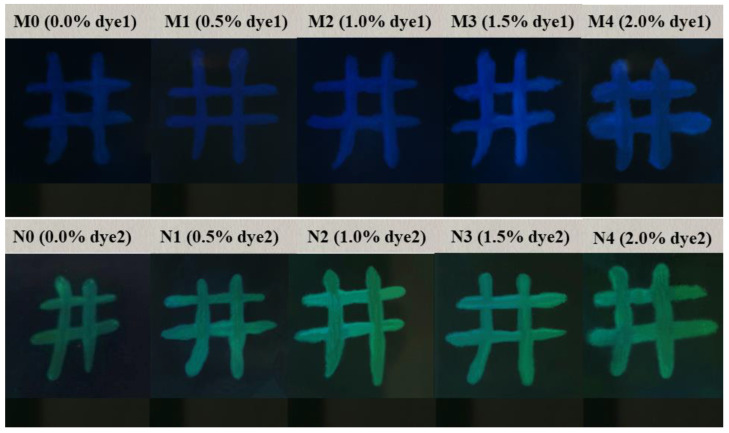
Physical images of PSBCLC films doped with different concentrations of dye1 and dye2.

**Figure 11:**
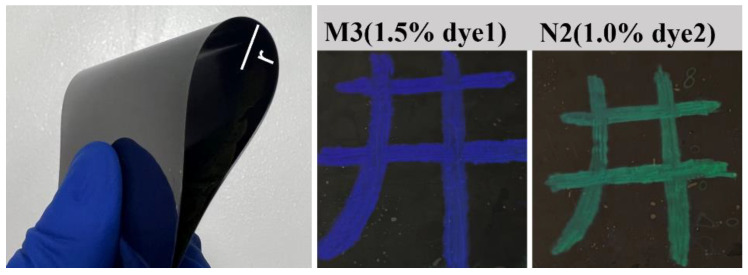
Physical images of samples M3 and N2 curvature bending tests.

Figure 10 shows the actual images of PSBCLC films doped with different concentrations of dye1 and dye2 in black and white PET LC cells. The BCLC molecules in the blue and green portions were in a planar state of selective reflection, while in the black portion, they were in a focal conic state of light scattering. It could be clearly seen that the contrast ratio and brightness of different concentrations of dye-doped PSBCLC films were significantly different. For dye1-doped PSBCLC films, the 1.5 wt% dye1-doped PSBCLC film had the highest contrast ratio and brightness. However, the difference in contrast ratio and brightness for different concentrations of dye2-doped PSBCLC films was not evident. Intuitively, 1.0 wt% dye2-doped PSBCLC film had a better performance, except for its lowest driving voltage, which was more energy-saving and environmentally friendly. The samples M3 and N2, which had the best properties, were selected for curvature bending tests, and the bending of the samples is shown in Figure 11. The radius of curvature of the film bending was 3 cm, and the performance of the samples did not change significantly after 20 repetitions, indicating that the prepared bistable cholesteric-phase liquid crystal films had good stability in the bending state (Table 2).

## 3. Experimental Section

### 3.1. Materials

**Figure 12:**
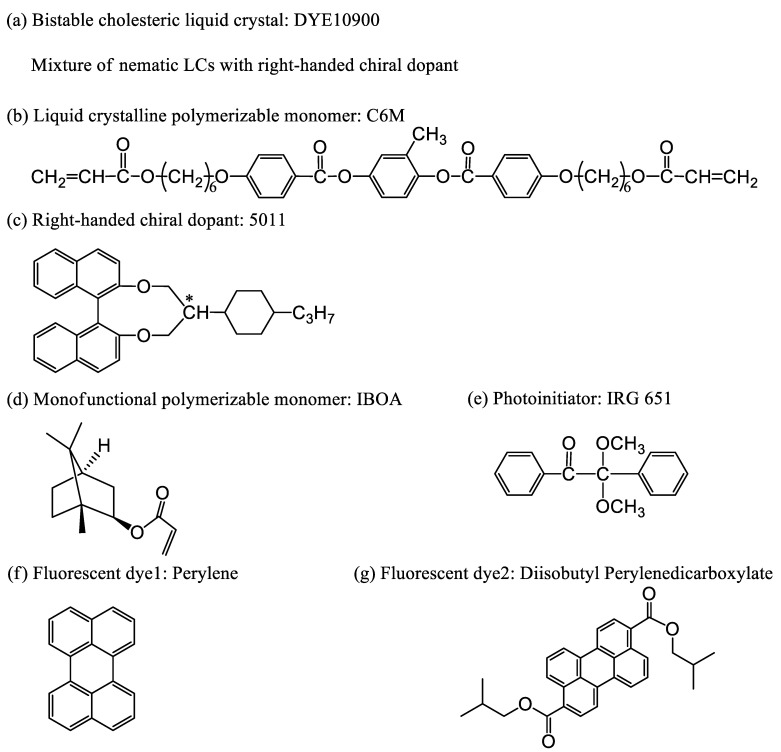
The chemical structures of the materials: (**a**) Bistable cholesteric liquid crystal: DYE10900; (**b**) Monomer: C6M; (**c**) The chiral dopant: R5011; (**d**) Monomer: IBOA; (**e**) Photoinitiator: IRG 651; (**f**) dye1; (**g**) dye2.

The host BCLC (DYE10900), provided by Shijiazhuang Chengzhi Yonghua Display Material Co., Ltd. (Shijiazhuang, China), exhibited a stable cholesteric phase at room temperature, with a clearing point of 66 °C. Its selective reflection band was between 520 nm and 590 nm, with the central reflection wavelength at 555 nm. The chiral dopant R5011, provided by Beijing Bayi Space LCD Technology Co., Ltd. (Beijing, China), was a right-handed chiral compound for adjusting the pitch of the cholesteric liquid crystal. The IRG651 (2,2-Dimethoxy-2-phenylacetophenone) was purchased from TCL Co., Ltd. (Huizhou, China) as a photoinitiator to initiate free radical polymerization of acrylic monomers under 365 nm UV lights. Isobornyl acrylate (IBOA) and 1,4-di-[4-(6-acryloyloxy) hexyloxy benzoyloxy]-2-methyl benzene (C6M), purchased from J&K Scientific Co., Ltd. (Beijing, China) and Beijing Kexin Jingyuan Electronics Co., Ltd. (Beijing, China), respectively, were selected as prepolymers of PSBCLCs. C6M was a liquid crystalline polymerizable monomer and could form a polymer network dissolved in a host liquid crystal, while IBOA was a non-liquid crystalline polymerizable monomer and used as a monomer diluent to reduce the viscosity of liquid crystal systems. The fluorescent dyes perylene and diisobutyl perylenedicarboxylate were provided by J&K Scientific Co., Ltd. and named dye1 and dye2, respectively. The fluorescence efficiencies of both fluorescent dyes were greater than 70%. The above experimental materials were used directly without any further modification, and the chemical structures of these materials are shown in Figure 12.

### 3.2. Preparing the Samples

In order to make the selective reflection bandwidth cover the emission wavelengths of fluorescent dyes, two new bistable cholesteric liquid crystals were prepared and named LC1 and LC2 using DYE10900 and R5011. As shown in Table 3, the different 5011 contents describe new LC-types in which LC1 and LC2 were perfectly matched to the emission wavelengths of dyes 1 and 2, respectively, and the fluorescent dyes were embedded in a super-twisted arrangement of LC cells, a cell configuration that allows for a more complete absorption of incident light, resulting in a darker off state. For the purpose of finding the best formula for polymer networks and building a better PSBCLC, the composites were prepared following the ratios according to Table 1, and then the composites were fully vibrated under ultrasonication until they formed a homogeneous mixture. When the optimal PSBCLC was successfully constructed, the different concentrations of fluorescent dyes were doped into PSBCLC according to Table 4. M0 and N0 with no fluorescent dyes were used as blank control samples. The fluorescent dyes were doped according to the following procedures and uniformly dispersed in the host liquid crystal: a certain amount of fluorescent dye was dissolved in dichloromethane, injected into the host liquid crystal, shaken fully to mix it completely, and then sonicated in a water bath at room temperature for one hour to make the fluorescent dye disperse evenly in the host liquid crystal. The formed uniform solution was placed in a vacuum drying oven at 40 °C for 48 h. Then, the dried samples were added to the other groups, according to Table 4, to make the final samples. Finally, the prepared samples were vibrated under ultrasonication thoroughly to form a homogeneous mixture. There were two kinds of liquid crystal cells for experimental measurement. One was a glass cell made of ITO-coated glasses with a 20 μm spacer to control the thickness. The other was a black and white ITO-coated PET cell, and the black PET film was coated with 5 μm PS microspheres to control the thickness of the liquid crystal. The liquid crystal gradually filled the glass cells due to the capillary action and was evenly stretched to fill the PET cells under external pressure.

### 3.3. Measurements

The transmission spectra and absorption spectra were obtained using a UV/VIS/NIR spectrophotometer (JASCO V-570), with the transmittance of an empty cell normalized to 100%. The LC optical textures of the samples were studied by polarizing optical microscopy (POM). The driving voltage of the LC molecule from the planar state to the focal conic state was measured and recorded using an AC contact-type voltage regulator. The contrast ratio (CR) was calculated from the transmittance in the planar state divided by the transmittance in the focal conic state at the center wavelength of the reflection bandwidth. The morphology of the polymer networks was observed by scanning electron microscopy (SEM). The above tests used glass liquid crystal cells. The fluorescence spectra of the PET samples were measured using a fluorescence spectrometer (BJCSKY F-280).

## 4. Conclusions

In conclusion, PSBCLC films doped with different concentrations of fluorescent dyes were prepared by constructing an optimal PSBCLC system. The optical properties, fluorescence intensities, and driving voltages of PSBCLC films with varying dye concentrations were evaluated. It was clear that PSBCLC films doped with 1.5 wt% perylene and PSBCLC films doped with 1.0 wt% diisobutyl had the highest contrast, 10.66 and 6.57, respectively, relatively low driving voltages, with values of 21 V and 25 V, as well as the highest fluorescence intensities. Compared with the PSBCLC film without dye doping, the PSBCLC film doped with 1.5 wt% perylenes and the PSBCLC film doped with 1.0 wt% diisobutyl had 5.61 and 1.6 higher contrasts and 6 and 8 V lower driving voltages, respectively. It not only greatly improved the utilization of incident lights, it also increased the contrast ratio and brightness by ingeniously combining fluorescent dyes with the PSBCLC. In addition, the PSBCLC films with different dye concentrations were explored to reduce the ACQ effect, which had guiding significance for related dye-doped studies. Finally, the brightness, high contrast ratio, and low-power PSBCLC films had a profound impact on reflective displays.

## Figures and Tables

**Table 1:** Chemical composition and weight percentage (wt%) of the PSBCLC samples.

Samples	IBOA	C6M	DYE10900	IRG651	UV Intensity (mW/cm^2^)
A	7.50	0.50	91.68	0.32	3.00
B	4.50	1.50	93.76	0.24	3.00
C1	4.00	1.00	94.80	0.20	1.00
C2	4.00	1.00	94.80	0.20	2.00 333
C3	4.00	1.00	94.80	0.20	3.00
C4	4.00	1.00	94.80	0.20	4.00
D	5.00	3.00	91.68	0.32	3.00

**Table 2:** Comparative analysis of existing literature on bistable CLCs with present work.

Reference	Materials	Dopants	Outcomes/Performance
M.-F. Sheng. (2021) [27]	DDLC Mixtures	Dichroic dye	Wearable Bistable Electrochromic Fabric Driving voltage: 9.7 V Temperature increased from 25 to 50 °C, the reflectance of redlight (approximately 620−740 nm) notably increased from 53.41 to 59.2.
C.-Y.Liu (2021) [28]	Nematic liquid crystal: HEF951 Chiral dopant S811	Chiral dopant ferroelectric liquid crystal 3C	Bistable cholesteric liquid crystal devices Focal conic to planar state: 36 ms Planar to focal conic state: 0.63 ms Average time: 18.31 ms Driving voltage: 5.5 V
G. Kocakülah (2021) [29]	Positive nematic LC: 5CB Chiral molecules: S811	Dichroic azo dye: Methyl Red	Electro-optical investigation of azo dye-doped CLC light shutter. Minimum transmittance value in the wavelength range of 475–525 nm, where the azo dye showed maximum absorption and had a significant effect on the transmittance of dye-doped CLC composite.
S.-W. Oh (2022) [30]	Positive nematic LC: ML-1104 Chiral molecules: R811 (for twist aligned (TA) and finger print (FP) cells)	Black dichroic dye: X12	In the initial state, electrically controlled birefringence (ECB), CLC, and FP cells were in the opaque state, with transmittance values of 36.07%, 19.05%, and 29.30%, respectively. The operating voltages for the transparent states of ECB, CLC, and FP cells were 17.6 V, 31.6 V and 4.2 V, respectively. The turn-on and turn-off times were found to be 6.52 ms and 44.6 ms, respectively, for FP cells.
Z.-M. He (2023) [31]	Nematic liquid crystal Chiral dopant: S811	Bifunctional polymerizable mesogenic monomer: C6M	Bistable PSCLC light shutter In the transparent state, high transmittance was presented so that the scene behind the sample could be seen clearly, while in the opaque state, the sample exhibited an intensive scattering state, and all objects behind it were blocked.
Present (reported here) work	Bistable cholesteric liquid crystal: DYE10900 Chiral dopant: R5011 Liquid crystalline polymerizable monomer: C6M Non-liquid crystalline polymer monomer: IBOA	Fluorescent dyes perylene and diisobutyl perylene dicarboxylate	1.5 wt% perylene, contrast: 10.66, driving voltage: 21 V 1.0 wt% diisobutyl, contrast: 6.57, driving voltage: 25 V

**Table 3:** Weight percentage and selective reflection bandwidth of two BCLCs.

Sample	DYE10900 (wt%)	R5011 (wt%)	Bandwidth (nm)	Center Wavelength (nm)
LC1	99.6	0.4	440–510	475
LC2	99.8	0.2	480–550	515

**Table 4:** Chemical composition and weight percentage (wt%) of different concentrations of fluorescent dye-doped PSBCLC samples.

Samples	IBOA	C6M	IRG651	LC		Fluorescent Dyes	
				LC1	LC2	Dye1	Dye2
M0	4.0	1.0	0.2	94.8	0.0	0.0	0.0
M1	4.0	1.0	0.2	94.3	0.0	0.5	0.0
M2	4.0	1.0	0.2	93.8	0.0	1.0	0.0
M3	4.0	1.0	0.2	93.3	0.0	1.5	0.0
M4	4.0	1.0	0.2	92.8	0.0	2.0	0.0
N0	4.0	1.0	0.2	0.0	94.8	0.0	0.0
N1	4.0	1.0	0.2	0.0	94.3	0.0	0.5
N2	4.0	1.0	0.2	0.0	93.8	0.0	1.0
N3	4.0	1.0	0.2	0.0	93.3	0.0	1.5
N4	4.0	1.0	0.2	0.0	92.8	0.0	2.0

## Data Availability

Not applicable.

## Sample Availability

Samples of the compounds are not available from the authors.
